# Higher intake of dietary dicarbonyl compounds is associated with lower incidence of type 2 diabetes: European Prospective Investigation into Cancer and Nutrition (EPIC)-InterAct case-cohort study

**DOI:** 10.1007/s00394-026-03904-0

**Published:** 2026-03-17

**Authors:** Kim Maasen, Ana-Lucia Mayen, Claudia Hana, Viktoria Knaze, Marleen M. J. van Greevenbroek, Simone J. P. M. Eussen, Charlotte Debras, Coen D. A. Stehouwer, Anne Tjønneland, Cecilie Kyrø, Daniel B. Ibsen, Christina C. Dah, Francesca Mancini, Nasser Laouali, Mariem Hajji, Matthias B. Schulze, Rashmita Bajracharya, Verena Katzke, Giovanna Masala, Fabrizio Pasanisi, Lorenzo Milani, Valeria Pala, Marta Farràs Mañé, Conchi Moreno-Iribas, Miguel Rodriguez-Barranco, Sandra Milena Colorado Yohar, Olatz Mokoroa, Keren Papier, Elisabete Weiderpass, Heinz Freisling, Nicholas J. Wareham, Nita G. Forouhi, Sofia Christakoudi, Philippe Vangrieken, Mazda Jenab, Casper G. Schalkwijk

**Affiliations:** 1 Department of Internal Medicine, CARIM School for Cardiovascular Diseases, Maastricht University Medical Centre, Maastricht University, Universiteitssingel 50, PO Box 616, 6229 ER Maastricht, The Netherlands; 2 Nutrition and Metabolism Branch, International Agency for Research on Cancer (IARC-WHO), Lyon, France; 3 Department of Nutritional Sciences, University of Vienna, Vienna, Austria; 4 Department of Epidemiology, CAPHRI Care and Public Health Research Institute/CARIM School for Cardiovascular Diseases, University Medical Centre, Maastricht, The Netherlands; 5 Danish Cancer Society Research Center, Copenhagen, Denmark; 6 Department of Public Health, University of Copenhagen, Copenhagen, Denmark; 7 Steno Diabetes Center Aarhus, Palle Juul-Jensens Vej 11, 8200 Aarhus N, Denmark; 8 Department of Public Health, Aarhus University, Bartholins Allé 2, 8000 Aarhus C, Denmark; 9 Department of Nutrition, Sports and Exercise, University of Copenhagen, Rolighedsvej 26, 1958 Frederiksberg C, Denmark; 10 MRC Epidemiology Unit, Institute of Metabolic Science, University of Cambridge School of Clinical Medicine, Cambridge Biomedical Campus, Cambridge, CB2 0QQ UK; 11 Université Paris-Saclay, UVSQ, INSERM “Exposome and Heredity” Team, CESP U1018, Gustave Roussy, Villejuif, France; 12 Department of Molecular Epidemiology, German Institute of Human Nutrition Potsdam-Rehbruecke, Nuthetal, Germany; 13 German Center for Diabetes Research (DZD), Munich-Neuherberg, Germany; 14 Institute of Nutritional Science, University of Potsdam, Nuthetal, Germany; 15 Department of Cancer Epidemiology, German Cancer Research Center (DKFZ), Heidelberg, Germany; 16 Institute for Cancer Research, Prevention and Clinical Network (ISPRO), Florence, Italy; 17 Dipartimento Di Medicina Clinica e Chirurgia, Federico II University, Naples, Italy; 18 Cancer Epidemiology Unit, Department of Medical Sciences, University of Turin, 10124 Turin, Italy; 19 Epidemiology and Prevention Unit, Fondazione IRCCS Istituto Nazionale Dei Tumori Di Milano, Milan, Italy; 20 Unit of Nutrition and Cancer, Epidemiology Research Program, Catalan Institute of Oncology (ICO), Bellvitge Biomedical Research Institute (IDIBELL), 08908 L’Hospitalet de Llobregat, Spain; 21 Instituto de Salud Pública y Laboral de Navarra, 31003 Pamplona, Spain; 22 Centro de Investigación Biomédica en Red de Epidemiología y Salud Pública (CIBERESP), 28029 Madrid, Spain; 23 Navarra Institute for Health Research (IdiSNA), 31008 Pamplona, Spain; 24 Escuela Andaluza de Salud Pública (EASP), 18011 Granada, Spain; 25 Instituto de Investigación Biosanitaria Ibs.GRANADA, 18012 Granada, Spain; 26 Department of Epidemiology, Murcia Regional Health Council, IMIB-Arrixaca, Murcia, Spain; 27 Research Group On Demography and Health, National Faculty of Public Health, University of Antioquia, Medellín, Colombia; 28 Ministry of Health of the Basque Government, Sub Directorate for Public Health and Addictions of Gipuzkoa, San Sebastián, Spain; 29 Epidemiology of Chronic and Communicable Diseases Group, BioGipuzkoa Health Research Institute, San Sebastián, Spain; 30 Cancer Epidemiology Unit, Nuffield Department of Population Health, University of Oxford, Oxford, UK; 31 Office of the Director, International Agency for Research On Cancer (IARC-WHO), Lyon, France; 32 MRC Epidemiology Unit, Institute of Metabolic Science, University of Cambridge School of Clinical Medicine, Cambridge Biomedical Campus, Cambridge, UK; 33 Department of Epidemiology and Biostatistics, School of Public Health, Imperial College London, St Mary’s Campus, Norfolk Place, London, W2 1PG UK; 34 Department of Inflammation Biology, School of Immunology and Microbial Sciences, King’s College London, London, UK

**Keywords:** Advanced glycation end products, Dietary dicarbonyl compounds, Food processing, Glycation, Methylglyoxal, Type 2 diabetes

## Abstract

**Purpose:**

Dicarbonyls are reactive precursors of advanced glycation end-products. They are formed during food processing, and endogenously in humans during glycolysis and lipid peroxidation. Higher plasma dicarbonyls, particularly methylglyoxal (MGO), promote insulin resistance and type 2 diabetes, but the association between dietary dicarbonyls intake and type 2 diabetes is unknown. This study examined the associations between dietary dicarbonyls and type 2 diabetes incidence.

**Methods:**

11,995 incident type 2 diabetes cases and a sub-cohort of 15,797 controls from the prospective multi-center European Prospective Investigation into Cancer and Nutrition (EPIC)-InterAct cohort were included. Intakes of three major dicarbonyls MGO, glyoxal [GO], and 3-deoxyglucosone [3-DG] were estimated at baseline using dietary questionnaires. Type 2 diabetes risk according to dietary dicarbonyl intake was estimated by multivariable-adjusted hazard ratios from Prentice-weighted Cox-regression analyses.

**Results:**

Higher intakes of MGO (sample-specific mean intake 3.4 ± 1.3 mg/d) and 3-DG (13.8 ± 10.5) were associated with lower incidence of type 2 diabetes (HR 0.92 [95% CI 0.90–0.95] for 1 SD higher MGO intake and 0.93 [0.90–0.95] for 1 SD higher 3-DG intake). No associations were observed for dietary GO.

**Conclusion:**

Participants who consumed more dietary dicarbonyls MGO and 3-DG had a lower risk to develop type 2 diabetes. This protective association contrasts with the harmful effects on type 2 diabetes risk reported for endogenously formed dicarbonyls.

**Supplementary Information:**

The online version contains supplementary material available at 10.1007/s00394-026-03904-0.

## Introduction

Dicarbonyls are highly reactive compounds and precursors of advanced glycation endproducts (AGEs) [1]. Dicarbonyls are inherently present in food items and their concentrations increase during food processing, mainly with heat treatment but also during fermentation [2]. Dicarbonyls are also formed endogenously in the body during glycolysis and lipid peroxidation. Three major dicarbonyl compounds are methylglyoxal (MGO), glyoxal (GO), and 3-deoxyglucosone (3-DG), of which MGO is the most reactive with a glycation potency that is 50,000 fold to that of glucose [1].

Higher concentrations of plasma MGO are thought to promote insulin resistance and type 2 diabetes [1, 3]. In contrast to endogenous dicarbonyls, the health consequences of intake of dicarbonyls from the diet are largely unknown. Data from a Dutch cross-sectional population-based study show that habitual consumption of some dicarbonyl compounds is positively correlated with their circulating concentrations and with higher tissue AGEs accumulation [4]. Other data from the same population suggest that higher dicarbonyl intakes are also associated with less low-grade inflammation [5] and with greater insulin sensitivity and lower prevalence of type 2 diabetes [6]. These findings suggest that dietary dicarbonyls contribute to body dicarbonyl and AGEs pools and appear to have metabolically beneficial effects.

Besides these studies, epidemiological data on the health effects of diet-derived dicarbonyls in humans is sparse and longitudinal studies are needed. Therefore, the current study aimed to examine the association between the intake of dietary dicarbonyls and incidence of type 2 diabetes, using data from the multicenter European Prospective Investigation into Cancer and nutrition (EPIC)-InterAct case-cohort study.

## Research design and methods

### Study design and population

**Fig. 1:**
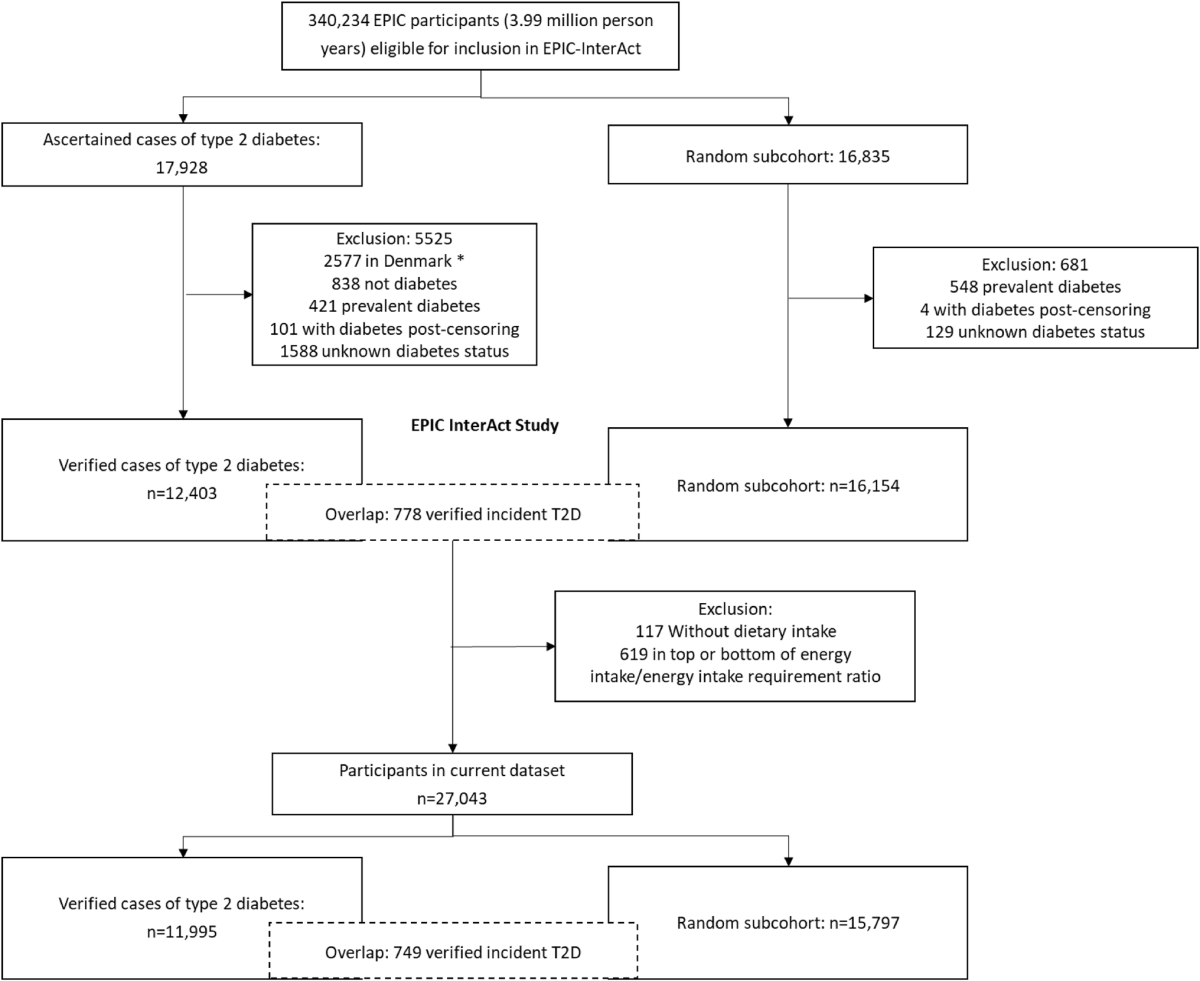
EPIC-InterAct case-cohort study design and flowchart of study participants.^*^A random sample of 2055 type 2 diabetes cases from Denmark was included after the exclusion of 2577 cases (due to logistic reasons)

The EPIC-InterAct study is a prospective case-cohort study nested within the EPIC cohort [7, 8]. In brief, the EPIC-InterAct study identified cases of type 2 diabetes among study participants from eight countries (France, Italy, Spain, the U.K., the Netherlands, Germany, Sweden, and Denmark). The study included 12,403 verified cases of incident type 2 diabetes occurring during 3.99 million person-years of follow-up of 340,234 EPIC participants eligible for InterAct, after exclusion of a random sample of individuals from Denmark due to logistic reasons (n = 2577 in cases group), individuals without ascertained type 2 diabetes in the cases group (n = 838), prevalent cases of type 2 diabetes at baseline (n = 421 in cases group and n = 548 in subcohort), incident diabetes after censoring (n = 101 in cases group and n = 4 in subcohort), and individuals with unknown diabetes status (n = 1588 in cases group and n = 129 in subcohort) (Fig. 1). As part of the EPIC-InterAct study, a random centre-stratified subcohort of 16,154 individuals was defined for comparative analyses. Individuals with incident diabetes during follow-up who were randomly selected into the subcohort while healthy (n = 778) were included as cases in the analyses. After exclusion of participants without dietary intake data (n = 117) and in the bottom and top percentile of the energy intake–to–requirement ratio (n = 619), a total of 11,995 type 2 diabetes cases and 15,797 participants in the subcohort were included in this analysis. All participants gave written informed consent, and the study was approved by the local ethics committees in the participating countries and by the Internal Review Board of the International Agency for Research on Cancer.

### Ascertainment and verification of cases of type 2 diabetes

Ascertainment of cases of incident type 2 diabetes included review of self-reported data, linkage to primary and secondary health care registers, use of diabetes medication (drug registers), hospital admission data, and mortality data, as described previously [8]. All self-reported cases were confirmed with at least one other independent source of information. Cases of type 2 diabetes in Denmark and Sweden were not ascertained by self-report but were identified through local and national diabetes and pharmaceutical registers, and were, therefore, considered verified.

### Assessment of dietary dicarbonyl intake

Dietary consumption over the 12 months before study enrolment was assessed by self-administered dietary questionnaires (except for Spain and Naples and Ragusa [Italy] where interview-administred questionnaires were used), mainly food frequency questionnaires (FFQs), developed and validated within each country as previously described [7]. Intakes of three major dicarbonyls (MGO, GO, and 3-DG) were estimated by combining the FFQs with a comprehensive database containing concentrations of MGO, GO, and 3-DG in 223 foods and drinks commonly consumed in a Western diet, quantified using the gold-standard ultra-performance liquid chromatography—tandem mass spectrometer (UPLC-MS/MS) [9]. Food items were all prepared according to regular preparation methods before quantification. For optimal matching to the food items covered by the FFQs used in EPIC, we additionally quantified MGO, GO, and 3-DG concentrations in 59 other food items that were missing in the database but commonly consumed in EPIC countries, using the same quantification method in the same laboratory (for a list of food items and their dicarbonyl concentrations, see Supplementary Table 7). Foods from the dicarbonyls database were matched to the food items in the FFQs by name and descriptors, particularly those pertaining to preparation and processing if applicable, in a similar manner as previously performed for the estimation of dietary AGE intake [10]. This included decomposing the recipes of food items to food ingredients, and applying multiplication of dicarbonyls concentrations with estimated factors for heat-treatment. In the matching, any generic or multi-ingredient food item from the FFQ was decomposed into more specific foods or ingredients based on country-specific recipes obtained from previous EPIC projects [11]. The EPIC dicarbonyls composition database was then generated and, in turn, used to obtain the daily intakes (mg/d) of MGO, GO, and 3-DG for each study participant. In a previous study, dicarbonyl intakes assessed by combining an FFQ and this dicarbonyl database, were positively associated with plasma concentrations of corresponding dicarbonyls [4].

### Measurement of other baseline characteristics

Information on baseline characteristics were obtained by questionnaires, including physical activity according to the Cambridge physical activity definition, highest attained educational level, alcohol intake at recruitment and during lifetime, smoking status, education, sociodemographic factors, medical history of prevalent hypertension, myocardial infarction, stroke or hyperlipidaemia, and – in women—menopausal status and ever hormone use was obtained from self-administered questionnaires [7]. Baseline weight, height, and waist circumference were collected by trained health professionals during a visit to a study centre, except in France and in the Oxford cohort, which obtained self-reported measures [7]. Body mass index (BMI) was calculated as weight (in kilograms) divided by square of height (in meters). Kidney function was assessed as estimated glomerular filtration rate (eGFR). eGFR was calculated using the Chronic Kidney Disease Epidemiology Collaboration (CKD-EPI) equation, using serum creatinine measured at baseline. Serum metabolic biomarkers (except in the Umeå centre [Sweden], where plasma was used) were measured at SHL (Stichting Ingenhousz Laboratory, Etten-Leur, Netherlands) from samples stored at − 196 °C ( − 80 °C in Umeå). These biomarkers included the liver function marker gamma glutamyl transferase [GGT], and an inflammatory biomarker (high-sensitivity C-reactive protein [hs-CRP]). Assays were performed using a Cobas® (Roche Diagnostics, Mannheim, Germany) assay on a Roche Hitachi Modular P analyser. A marker of glycaemic control, haemoglobin A1c (HbA1c), was measured at Stichting Ingenhousz Laboratory in the erythrocyte fraction from samples stored at − 196 °C ( − 80 °C in Umeå) using the Tosoh-G8 analyser (Tosoh Bioscience, Japan).For a more detailed description see reference [8].

### Statistical analysis

Baseline characteristics were displayed for the non-cases of the randomly selected subcohort of EPIC-InterAct, stratified according to quintiles of energy adjusted residuals of MGO, GO, and 3-DG intake. Dietary intakes of MGO, GO, 3-DG, and main food groups were displayed for overall subcohort and stratified by country.

Dietary intakes of MGO, GO, and 3-DG (in mg/day) were adjusted for total energy intake. For energy adjustment, we computed standardized residuals of each dietary dicarbonyl by regressing the dietary dicarbonyls on total energy intake and centre. These energy adjusted residuals of MGO, GO and 3-DG were each standardized by dividing the variables by their standard deviation for comparison. The dietary dicarbonyls were analysed individually, because they are thought to exert different biological effects [12].

#### Associations of dietary MGO, GO, and 3-DG intakes with risk of type 2 diabetes

The associations of energy-adjusted MGO, GO, and 3-DG intakes with risk of type 2 diabetes were estimated with hazard ratios (HRs) and 95% confidence intervals (CIs) using Cox proportional hazards Models, adapted to case-cohort designs according to the Prentice-weighted method with robust standard errors and with attained age as the underlying time scale [13]. Entry time was age at recruitment and exit time was either age at diagnosis, death or censoring date (lost to or end of follow-up). Participants with missing values were included in analyses and respective variables were coded with a missing value indicator (education n = 236, menopausal status (men/missing value) n = 12,954, hormone use (men/missing value) n = 14,899, and hypertension n = 5,246), because restriction to only participants with complete data may introduce selection bias.

Our statistical analysis strategy was based on multiple models. Model 1 was stratified by sex, centre, and age at recruitment (in 5-year categories). Model 2 was identical to the first model but with additional adjustments for BMI (continuous, kg/m^2^); height (continuous, cm); smoking status (never, former, current, unknown); alcohol intake (continuous, grams per day); physical activity as defined by the Cambridge Index (inactive, moderately inactive, moderately active, active, and missing); education (none, primary school, technical/professional school, secondary school, longer education, and missing); total energy intake (continuous, kcal/d); menopausal status (premenopausal, postmenopausal, perimenopausal, surgical postmenopausal, men/missing); and ever use of hormone before menopause (yes, no, men/missing).

We performed several sensitivity analyses. First, we accounted for potential reverse causality by excluding people with HbA1c greater than or equal to 6.5% (48 mmol/mol) at baseline or those confirmed as having type 2 diabetes within the first two years or first four years after baseline. Second, we excluded participants with prevalent cardiovascular disease (i.e., hypertension, myocardial infarction, and/or stroke at baseline), because this might have led to dietary and/or lifestyle changes. Third, we additionally adjusted model 2 for diet quality (Model 2+ Mediterranean Diet Score), to explore whether the associations could be explained by a generally healthier diet. The Mediterranean Diet Score indicates higher intakes of amongst others vegetables, legumes, fruit and nuts/seeds, cereals, fish and seafood, olive oil [14]. Because these food groups are also sources of dietary dicarbonyls, adjusting for Mediterranean diet index may be overadjustment and therefore was not added in the main analyses. Fourth, we additionally adjusted Model 2 for soft drinks intake, as soft drinks intake is associated with risk of type 2 diabetes [15], but are also a source of dicarbonyls (median concentrations soft drinks, MGO: 0.24 mg/L, GO: 0.3 mg/L, 3-DG: 2.5 mg/L) [9] and was therefore not included in the main model. Fifth, to explore whether the association was via a lower low-grade inflammation, prompted by our previous observation of an association between dietary MGO intake and less low-grade inflammation [5], we repeated the analyses after additional adjustment of model 2 for the inflammatory biomarker C-Reactive protein (hsCRP) (ng/ml). Sixth, we additionally adjusted model 2 for waist circumference on top of adjustment for BMI (using residuals of waist circumference regressed by BMI to prevent multicollinearity when adding both BMI and waist to the model). Seventh, we mutually adjusted model 2 for intakes of the two other dietary dicarbonyls by adding them to the model, to examine if associations were independent of the other dietary dicarbonyls.

Interaction was tested with sex, BMI, waist circumference, smoking status, low-grade inflammation, kidney function and liver function, using the Wald test for the interaction term. In case of significant interaction with one of these interaction terms (*P* < 0.1), the fully adjusted analyses (Model 2) were repeated in subgroups according to the corresponding variable. For BMI, we used categories defined by the World Health Organisation (WHO) as follows ‘underweight’ = BMI below 18.5; ‘normal weight’ = 18.5–24.9; ‘pre-obesity’ = 25.0–29.9; ‘obesity Class I’ = 30.0–34.9; ‘obesity Class II’ = 35.0–39.9; ‘obesity Class III’ = above 40) [16].

To evaluate the shape and linearity of associations for continuous intakes of dicarbonyl compounds (g/day), three-knot restricted cubic spline models were fitted at Harrell's default percentiles (i.e. 10th, 50th and 90th) in combination with a Wald-type test for nonlinearity [17], based on model 2. HRs are presented for MGO, GO, and 3-DG intake as continuous exposure (per one SD higher intake) and as country-specific quintiles of dietary dicarbonyls, built according to the intake levels of non-cases in the sub-cohort. In case of linearity, conclusions were based on the continuous model, and in case of non-linearity on the categorical model. We also analysed linear trends across MGO, GO, and 3-DG categories using each quintile of intake as a continuous exposure variable (categories 1–5, p-for-trend).

In addition, we performed subgroup analyses by sex, age (< 50, 50–65 and ≥ 65 years) and country. To explore differences between countries, we fitted model 2 separately in each country and pooled risk estimates using random effects meta-analyses and assessed heterogeneity of associations across countries using the I-squared measures proportion of total variability due to between-study (country) heterogeneity [18].

The Cox proportional hazards assumption that regression coefficients are similar over time was checked by including an interaction term between the exposures and the underlying timescale in the Cox model (data not shown).

#### Contribution of main food groups to the strength of the associations

To explore the extent of the association that could be explained by intake of certain food groups, model 2 was additionally adjusted by intake of food groups that are the top-5 contributors to intake of each dicarbonyl (added to the model in turn; MGO: coffee (31%); cereals (21%); vegetables (10%); meat (8%); cakes and biscuits (6%); GO: cereals (22%); fruit, nuts and seeds (21%); vegetables (16%); fruit and vegetable juices (5%); coffee (5%); 3-DG: cereals (21%); fruit, nuts and seeds (17%); sugar and confectionary (16%);beer (9%); cakes and biscuits (9%)). Information about food sources for the dietary dicarbonyls was derived from the full EPIC-cohort, from which the EPIC-InterAct case-cohort was selected.

All statistical analyses were performed in Stata 14 (Statacorp, College Station, TX).

## Results

### Characteristics of the study population

Our analysis of this case-cohort study consisted of 11,995 incident type 2 diabetes cases and a sub-cohort of 15,797 participants (including 749 incident type 2 diabetes cases) with a median [IQR] follow-up time of 10.9 [7.2–12.7] years. Dicarbonyl intakes (mg/day, mean ± SD) in non-cases in the sub-cohort were 3.4 ± 1.3 for MGO, 2.9 ± 0.98 for GO, and 13.8 ± 10.5 for 3-DG (Supplementary Table 1). Individuals in the highest quintile of MGO intake had higher intakes of coffee, vegetables, fruits, nuts and seeds, fruit and vegetable juices, and lower intakes of dairy and soft drinks (Table 1). Individuals with a higher MGO intake also had higher GO and 3-DG intakes. They were more often current smokers compared to those in the lowest quintile but did not differ in the other health characteristics measured. Individuals in the highest quintile of GO intake were more often women than those in the lowest quintile. They had a lower intake of alcohol and were more physically active and less often current smokers. Individuals with higher GO intakes had higher intakes of coffee, vegetables, fruits, nuts and seeds, and fruit and vegetable juices and soft drinks, a lower intake of meat. Individuals in the highest quintile of 3-DG intake were more often men, had a higher alcohol intake, were somewhat more physically active and had a higher education compared to those in the lowest quintile. They had higher intakes of vegetables, fruits, nuts and seeds, but also a higher intake of sugar and confectionary, fruit and vegetable juices, and beer, and lower intakes of cereals, meat, potatoes, and dairy.

**Table 1:** Baseline characteristics of the EPIC-InterAct subcohort population, according to quintiles of residuals of dietary dicarbonyls, country-specific quintiles based on non-cases (n = 15,797)

Characteristics	MGO					GO					3-DG				
	Q1	Q2	Q3	Q4	Q5	Q1	Q2	Q3	Q4	Q5	Q1	Q2	Q3	Q4	Q5
Participants (n)	3162	3150	3156	3160	3169	3161	3146	3175	3142	3173	3178	3144	3175	3160	3140
Case status (incident diabetes)															
Non-cases (n)	3013	3009	3011	3009	3006	3013	3009	3011	3009	3006	3013	3009	3011	3009	3006
Cases (n)	149	141	145	151	163	148	137	164	133	167	165	135	164	151	134
MGO consumption, mg/day^a,b,c^	2.2 ± 0.75	2.9 ± 0.82	3.3 ± 1.0	3.7 ± 1.1	4.4 ± 1.4	2.9 ± 1.2	3.2 ± 1.2	3.3 ± 1.3	3.5 ± 1.3	3.8 ± 1.4	3.0 ± 1.3	3.2 ± 1.2	3.3 ± 1.3	3.4 ± 1.3	3.7 ± 1.3
GO consumption, mg/day^a,b,c^	2.5 ± 0.90	2.8 ± 0.91	3.0 ± 0.94	3.1 ± 0.97	3.2 ± 0.96	2.0 ± 0.65	2.6 ± 0.70	2.9 ± 0.70	3.2 ± 0.77	3.8 ± 0.96	2.6 ± 0.95	2.8 ± 0.93	3.0 ± 0.95	3.1 ± 0.94	3.2 ± 0.95
3-DG consumption, mg/day^a,b,c^	11.2 ± 8.0	13.3 ± 9.5	14.2 ± 9.8	14.6 ± 11.4	15.8 ± 13.5	11.2 ± 8.0	12.7 ± 8.0	13.3 ± 8.4	14.9 ± 11.7	17.1 ± 14.8	8.0 ± 4.3	10.3 ± 4.8	12.3 ± 5.4	15.0 ± 7.0	23.6 ± 17.6
Age at recruitment, yrs	53 ± 9.8	53 ± 9.3	52 ± 9.1	52 ± 9.1	52 ± 8.5	51.5 ± 9.1	52.2 ± 9.2	52.5 ± 9.1	52.8 ± 9.2	53.0 ± 9.2	53 ± 9.1	52 ± 9.3	52 ± 9.3	52 ± 9.2	53 ± 8.8
BMI, kg/m^2^	26 ± 4.4	26 ± 4.2	26 ± 4.1	26 ± 4.1	26 ± 4.2	26 ± 4.3	26 ± 4.0	26 ± 4.1	26 ± 4.2	26 ± 4.3	26 ± 4.5	26 ± 4.2	26 ± 4.1	26 ± 4.1	26 ± 4
Height, cm	166 ± 9.3	166 ± 9.2	167 ± 9.3	166 ± 9.4	166 ± 9.2	167 ± 9.4	167 ± 9.5	166 ± 9.3	166 ± 9.3	165 ± 8.8	165 ± 9.1	166 ± 9.4	166 ± 9.3	167 ± 9.3	167 ± 9.3
Women, %	64	60	60	62	66	53	56	63	66	73	67	65	63	59	58
Dietary variables															
Total energy, kcal/day	2052 ± 721	2196 ± 654	2205 ± 632	2165 ± 592	2067 ± 538	2083 ± 741	2179 ± 679	2150 ± 619	2148 ± 576	2125 ± 528	2117 ± 763	2140 ± 672	2146 ± 608	2156 ± 579	2127 ± 516
Coffee, g/day	104 ± 123	253 ± 200	371 ± 285	480 ± 350	711 ± 517	350 ± 348	374 ± 367	392 ± 389	399 ± 395	404 ± 416	391 ± 411	377 ± 369	396 ± 387	378 ± 372	378 ± 380
Vegetables, g/day	157 ± 99	180 ± 108	184 ± 116	194 ± 129	205 ± 137	132 ± 89	163 ± 97	183 ± 106	201 ± 116	241 ± 151	171 ± 111	180 ± 113	189 ± 121	190 ± 122	190 ± 129
Legumes, g/day	17 ± 28	19 ± 31	18 ± 30	18 ± 29	16 ± 25	16 ± 28	18 ± 29	18 ± 29	18 ± 29	18 ± 28	18 ± 30	18 ± 30	18 ± 30	18 ± 28	16 ± 26
Fruit, nuts, seeds, g/day	209 ± 156	237 ± 183	250 ± 190	256 ± 207	256 ± 207	106 ± 83	178 ± 114	228 ± 131	281 ± 156	414 ± 256	220 ± 182	244 ± 191	249 ± 203	245 ± 181	250 ± 191
Soft drinks, g/day	85 ± 194	75 ± 160	77 ± 154	69 ± 144	68 ± 155	65 ± 145	76 ± 157	69 ± 139	80 ± 171	81 ± 194	74 ± 180	71 ± 157	80 ± 166	80 ± 169	68 ± 136
Cereals, g/day	209 ± 119	227 ± 117	226 ± 116	224 ± 114	211 ± 110	206 ± 126	223 ± 116	226 ± 116	224 ± 109	219 ± 109	226 ± 139	224 ± 118	221 ± 111	217 ± 101	210 ± 104
Sugar and confectionary, g/day	38 ± 35	39 ± 32	39 ± 32	37 ± 30	36 ± 32	35 ± 32	37 ± 32	38 ± 32	40 ± 33	39 ± 34	33 ± 31	37 ± 31	38 ± 32	40 ± 33	41 ± 35
Cakes and biscuits, g/day	38 ± 46	43 ± 49	45 ± 47	45 ± 45	42 ± 41	35 ± 42	44 ± 47	44 ± 46	47 ± 48	43 ± 43	36 ± 43	43 ± 48	43 ± 45	47 ± 48	44 ± 45
Meat and meat products, g/day	103 ± 61	112 ± 62	114 ± 61	114 ± 60	109 ± 57	120 ± 66	119 ± 64	112 ± 59	107 ± 56	95 ± 53	118 ± 68	114 ± 60	111 ± 59	108 ± 58	101 ± 55
Fish and shellfish, g/day	26 ± 26	29 ± 29	30 ± 31	30 ± 31	30 ± 32	27 ± 29	29 ± 29	29 ± 29	30 ± 31	30 ± 32	29 ± 30	29 ± 30	29 ± 30	29 ± 30	29 ± 30
Egg and egg products, g/day	19 ± 19	19 ± 18	19 ± 17	18 ± 17	18 ± 17	20 ± 20	19 ± 18	19 ± 17	18 ± 16	17 ± 16	20 ± 19	19 ± 18	19 ± 17	18 ± 17	17 ± 16
Fruits and vegetable juices, g/day	51 ± 93	61 ± 107	63 ± 114	69 ± 132	67 ± 126	28 ± 53	42 ± 69	53 ± 83	71 ± 109	115 ± 188	39 ± 81	55 ± 104	65 ± 118	67 ± 113	84 ± 147
Potatoes, g/day	97 ± 76	99 ± 77	104 ± 80	101 ± 80	91 ± 68	98 ± 77	102 ± 77	102 ± 78	97 ± 75	94 ± 76	103 ± 83	102 ± 78	100 ± 76	97 ± 75	91 ± 70
Dairy, g/day	356 ± 267	350 ± 244	346 ± 242	326 ± 223	311 ± 219	338 ± 269	341 ± 242	345 ± 235	337 ± 232	327 ± 220	371 ± 275	346 ± 240	336 ± 233	325 ± 224	310 ± 220
Beer, g/day	76 ± 216	103 ± 252	113 ± 251	110 ± 243	117 ± 277	177 ± 393	120 ± 250	93 ± 201	77 ± 167	52 ± 124	31 ± 70	50 ± 76	76 ± 140	121 ± 224	242 ± 447
Mediterranean diet score, %^d^															
Low	29	27	28	27	26	42	32	27	22	14	34	29	26	24	25
Medium	47	45	42	43	44	43	44	44	44	47	43	43	44	46	46
High	24	28	30	30	30	15	24	30	34	39	24	28	30	30	30
Alcohol at recruitment, g/day	12 ± 18	14 ± 20	15 ± 19	13 ± 17	12 ± 17	20 ± 26	15 ± 19	12 ± 16	10 ± 14	7.5 ± 11	7.8 ± 13	11 ± 16	13 ± 17	15 ± 19	18 ± 24
Lifetime alcohol intake, %															
Never drinkers	9	6	6	6	7	6	6	7	7	9	10	7	6	5	7
Former drinkers	6	5	4	5	6	4	4	5	6	7	8	5	4	5	5
Drinkers only at recruitment	6	3	4	4	4	4	4	4	4	5	5	4	4	4	4
Lifetime drinkers	55	62	61	62	57	63	62	59	58	55	53	59	61	62	62
Unknown	24	24	24	23	25	24	24	25	25	24	23	25	25	25	23
Physical activity, %															
Inactive	26	23	22	21	23	26	23	22	21	22	26	23	22	22	21
Moderately inactive	33	34	34	34	34	34	34	36	33	32	34	34	35	33	34
Moderately active	22	23	23	22	22	22	22	22	23	22	21	22	22	24	23
Active	19	19	20	22	19	17	19	20	21	22	18	19	21	20	21
Missing	1	1	1	1	1	1	1	1	1	1	1	1	1	1	1
Smoking status, %															
Never	54	49	47	44	39	36	44	48	51	54	45	48	47	48	44
Former	25	29	26	28	27	26	28	27	28	27	25	27	27	27	30
Current	20	22	26	28	33	38	28	24	21	18	30	24	25	24	25
Unknown	1	1	1	1	1	1	1	1	1	1	1	1	1	1	1
Education, %															
None	10	8	7	7	7	7	8	8	8	7	9	8	7	7	7
Primary completed	33	31	32	33	35	34	33	33	32	32	35	33	32	31	32
Technical/ professional	21	22	24	24	24	22	21	23	24	24	23	22	22	23	23
Secondary	16	15	16	15	14	15	15	15	15	15	15	15	15	16	15
Tertiary	20	23	20	20	19	19	21	20	20	21	16	20	22	21	23
Missing	1	2	2	2	2	2	2	2	2	2	2	1	2	2	2
Hyperlipidaemia, %															
Yes	16	18	19	20	19	17	18	18	18	21	17	16	18	20	21
Hypertension, %															
Yes	19	20	18	17	17	17	18	19	20	18	19	18	18	19	18
Menopausal status, %															
Premenopausal	21	18	20	21	21	20	18	21	20	22	21	22	21	20	18
Postmenopausal	31	29	27	28	31	22	26	30	31	36	33	29	29	27	28
Perimenopausal	10	10	10	11	12	10	8,9	11	11	12	11	12	10	10	10
Surgical postmenopausal	3	2	2	2	3	2	2	2	3	3	3	2	2	2	2
Men/missing	36	40	40	38	34	47	44	37	34	27	33	35	37	41	42
Ever hormone use, %															
Yes	14	14	13	14	15	11	13	14	15	17	15	14	15	14	12

Residuals were computed by a linear regression of the intake of dicarbonyls, energy and center

^a^Mean ± SD, all such values

^b^Crude intake in mg/day, not adjusted for energy intake

^c^MGO intake was highest in Denmark (4.6 mg/day, Supplementary Table 1) and lowest in Italy and Spain (2.5 mg/day). GO intake was highest in the United Kingdom (3.5 mg/day respectively), and lowest in Italy and Spain (2.6 mg/day). 3-DG intake was highest in the United Kingdom (23 mg/day) and lowest in Spain (8.5 mg/day)

^d^Categorical variable, modified relative Mediterreanean diet score used. Calculated as in https://doi.org/10.1093/aje/kwp282

### Associations of MGO, GO, and 3-DG intake with risk of type 2 diabetes

**Fig. 2:**
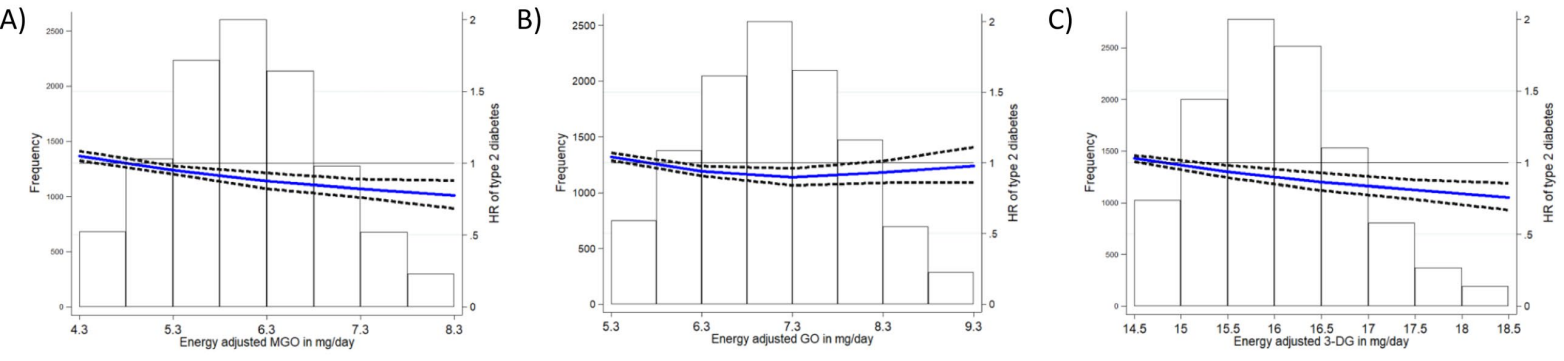
Associations between dicarbonyl compounds consumption (energy-adjusted mg/day) and incident type 2 diabetes. Blue line displays the hazard ratio, dashed lines display the 95% confidence interval

A higher intake of MGO was associated with a lower incidence of type 2 diabetes in both Model 1 (crude) and Model 2 (multivariable adjusted models; HR per SD increase in intake of energy adjusted MGO residuals = 0.92 [95% CI 0.90–0.95]; HR for highest vs. lowest quintiles = 0.79 [0.72–0.87], P-trend < 0.001; Table 2). The linear inverse risk association for MGO was confirmed with restricted cubic spline models (*P* for non-linearity = 0.07, Fig. 2). Results for country specific associations by random effects meta-analyses are shown in Supplementary Fig. 2, Supplementary Table 1, and described in the supplementary file 1.

**Table 2:** HRs (95% CIs) for incident type 2 diabetes according to dicarbonyl intake, EPIC InterAct (n = 27,043)

	Dicarbonyl intake						P-trend
	Continuous (per 1 SD increase)	Q1	Q2	Q3	Q4	Q5	
MGO intake in mg/day (median [IQR])^a^	3.12 [2.42–4.05]	2.16 [1.74–2.68]	2.84 [2.38–3.43]	3.20 [2.70–3.94]	3.57 [2.92–4.34]	4.33 [3.41–5.38]	
n cases/N	11,995 / 27,043	2,628 / 5,641	2,435 / 5,444	2,383 / 5,394	2,236 / 5,245	2,313 / 5,319	
Model 1	0.96 (0.93, 0.98)	1 (reference)	0.93 (0.88, 0.99)	0.93 (0.88, 0.98)	0.88 (0.84, 0.94)	0.94 (0.89, 0.99)	0.001
Model 2	0.92 (0.90, 0.95)	1 (reference)	0.91 (0.83, 1.00)	0.87 (0.79, 0.95)	0.82 (0.74, 0.90)	0.79 (0.72, 0.87)	< 0.001
GO intake in mg/day (median [IQR])	2.79 [2.22–3.45]	1.97 [1.61–2.44]	2.48 [2.11–2.97]	2.79 [2.37–3.29]	3.10 [2.64–3.63]	3.67 [3.15–4.35]	
n cases/N	11,995 / 27,043	2,757 / 5,770	2,392 / 5,401	2,361 / 5,372	2,161 / 5,170	2,324 / 5,330	
Model 1	0.91 (0.90, 0.95)	1 (reference)	0.89 (0.84, 0.94)	0.88 (0.83, 0.94)	0.83 (0.79, 0.88)	0.89 (0.84, 0.94)	< 0.001
Model 2	0.97 (0.94, 1.00)	1 (reference)	0.93 (0.85, 1.02)	0.92 (0.84, 1.01)	0.83 (0.75, 0.92)	0.94 (0.86, 1.04)	0.037
3-DG intake in mg/day (median [IQR])	11.5 [7.80–16.8]	7.27 [5.10–10.2]	9.61 [7.00–12.8]	11.7 [8.53–15.5]	14.0 [10.0–18.5]	19.8 [14.0–28.1]	
n cases/N	11,995 / 27,043	2,831 / 5,844	2,431 / 5,440	2,432 / 5,443	2,227 / 5,236	2,074 / 5,080	
Model 1	0.87 (0.84, 0.89)	1 (reference)	0.89 (0.84, 0.94)	0.87 (0.82, 0.91)	0.81 (0.76, 0.85)	0.75 (0.71, 0.79)	< 0.001
Model 2	0.93 (0.90, 0.95)	1 (reference)	0.94 (0.86, 1.03)	0.99 (0.90, 1.08)	0.85 (0.77, 0.93)	0.80 (0.73, 0.89)	< 0.001

Model 1: crude Model, stratified by age, sex and center

Model 2: Adjusted for energy intake, educational level, BMI height, physical activity, smoking status, alcohol (continuous, grams/day), menopause, hormone use

Dicarbonyl intakes (when used as main independent variables) are standardized energy adjusted residuals

^a^median [IQR] are crude intakes, so not energy adjusted residuals. Quintiles are country-specific and based on non-cases

*MGO* Methylglyoxal, *GO* Glyoxal, *3-DG* 3-Deoxyglucosone

The association between GO intake and type 2 diabetes risk was non-linear (P for non-linearity = 0.002, Fig. 2). Compared to the lowest quintile, any quintile of GO intake was inversely associated with type 2 diabetes risk, but the association was only statistically significant in the fourth quintile (HR 0.83 [0.75, 0.92], Model 2, Table 2).

Similar to associations observed with MGO, 1 SD higher 3-DG intake was associated with a 7% lower incidence of type 2 diabetes after adjustment for confounders (HR 0.93 [0.90, 0.95], Model 2, Table 2). Analysing 3-DG intake by quintile gave similar results (highest vs. lowest quintile HR 0.80 [0.73, 0.89], P-trend < 0.001). Assessment of the shape of the dose–response relationship with restricted cubic splines showed a linear inverse association (P for non-linearity = 0.12, Fig. 2).

### Sensitivity analyses

The associations between higher MGO and 3-DG intakes and lower risk of type 2 diabetes were robust over all sensitivity analyses, such as additional adjustment for adherence to the Mediterranean diet, excluding individuals with an HbA1c of 6.5% or higher at baseline, those with type 2 diabetes within the first two or four years after baseline, or those with prevalent cardiovascular diseases (Supplementary Table 2).

### Interaction analyses

Interaction terms of dietary dicarbonyls with sex, BMI, waist, smoking, inflammation, kidney function and liver function were added to evaluate whether the associations differed between men and women, with varying levels of adiposity, inflammation, kidney function (involved in the excretion of dicarbonyls) or liver function (potentially involved in the metabolism of dicarbonyls), and over smoking status. The associations of MGO and 3-DG intakes and type 2 diabetes risk were not modified by any of these variables (MGO: p-interaction 0.10–0.81; 3-DG: p-interaction 0.05–0.73, Supplementary Table 3). The association between dietary GO intake and type 2 diabetes risk was modified by sex and by BMI (p-interaction 0.01 and 0.04, respectively). After stratification by sex, the association between GO intake and type 2 diabetes risk was stronger in men than in women (Supplementary Table 4). After stratification by BMI, the association was stronger in individuals with a lower BMI (Supplementary Table 5).

### Contribution of main food groups to the strength of the associations

To explore whether the observed associations were driven by any food groups in particular, we alternately adjusted Model 2 for intake from each of the food groups in the top 5 of MGO, GO, or 3-DG intake (corresponding to the dicarbonyl used as the predictor).

The association between MGO intake and type 2 diabetes risk was mainly driven by coffee intake, because additional adjustment for coffee attenuated this association (Model 2: HR 0.92 [0.90, 0.95]; Model 2+ coffee intake: HR 0.98 [95% CI 0.93–1.03]). Besides this, the associations remained similar after additional adjustment for food groups (Supplementary Table 6).

## Discussion

In this prospective case-cohort study with 11,995 incident type 2 diabetes cases from eight European countries followed-up for a median of 11 years, we observed lower risk of type 2 diabetes in individuals with a higher habitual dietary intake of MGO and 3-DG. A similar, although not linear, trend was seen for GO which did not remain significant upon adjustment for potential confounders. The observed inverse risk association between dietary MGO intake and type 2 diabetes seemed to be driven mainly by coffee consumption, a major source of MGO in the diet. These associations were robust over a range of sensitivity analyses, such as exclusion of cases in the first two or four years of follow-up or with an HbA1c ≥ 6.5% to minimize reverse causation and were not due to a generally healthier diet because additional adjustment by the Mediterranean diet score, a dietary pattern recognized for its general health effects, did not change our results.

These results corroborate and expand on the earlier-reported cross-sectional associations of higher dietary intakes of MGO and 3-DG, but not GO, with less low-grade inflammation [5] and with greater insulin sensitivity and lower prevalence of type 2 diabetes [6]. In line, we previously reported an inverse association between dietary dicarbonyl intakes and incident cardiovascular diseases in the same cohort, which may be related to the lower incidence of type 2 diabetes observed in the current study, since cardiovascular diseases are a major consequence of type 2 diabetes [19]. The biological mechanisms underlying the association between dietary dicarbonyl and lower risk of type 2 diabetes is currently unknown. The association between dietary MGO intake and lower risk of type 2 diabetes reported here did not seem to be modulated via differences in inflammation because results were unchanged after adjustment for hs-CRP. Several other potential mechanisms are conceivable. Firstly, exposure to dicarbonyls from the diet might exert local effects in the gastro-intestinal tract, for example via modulation of the gut microbiome, as has previously been reported for AGEs [20, 21]. At the same time, a previous study reported an association between higher dietary dicarbonyl intakes and higher concentrations of corresponding dicarbonyls in the circulation [4]. This observation suggests that dicarbonyls from food are, at least partly, absorbed in the gastro-intestinal tract and enter the circulation, hence contributing to the circulating pool of dicarbonyls. Higher circulating levels of dicarbonyls are associated with adverse health outcomes (as reviewed in [1]), but it is conceivable that a small but consistent flux into the plasma pool via exogenous intake, may have beneficial effects, for example through upregulation of the anti-oxidant system and/or the glyoxalase system, a major pathway in the detoxification of MGO [22].

In line with our current findings, several recent animal studies have reported beneficial effects of long-term (12–18 months) exposure to oral MGO via drinking water. Beneficial effects included improvement in antioxidant systems [23] and slightly increased survival rate [24], despite elevated MGO concentrations in the circulation. Similarly, low doses of exogenous MGO have been shown to promote increased lifespan in experimental models with *Drosophila* and C. *Elegans* [3, 25]. The hypothesized mechanism behind these beneficial effects is an MGO-induced upregulation of defence mechanisms against oxidative stress, involving the KEAP1-Nrf2 pathway [22, 26, 27]. Nrf2 is a transcription factor and a key regulator in oxidative stress, amongst others leading to a higher production of the detoxifying enzyme of MGO, glyoxalase-1. Based on these findings, a hormetic effect of MGO has been proposed, which implies a beneficial response to low-dose exposure with stressors that are harmful at a higher dose [22].

In contrast, other animals studies report adverse effects of oral administration of MGO which suggest that it may promote type 2 diabetes, including impaired insulin sensitivity, greater insulin resistance [3, 28, 29], impaired glucose tolerance [3, 30], and reduced insulin secretion by the pancreatic β-cells [30]. However, translation from these animal studies to humans is difficult, because these experiments were often conducted with very high concentrations of MGO, outside of the physiological range. Moreover, the effects of MGO via drinking water might be different than MGO from the diet, where it could interact with the many other food constituents present, affecting absorption.

Our current findings challenge the belief that dietary dicarbonyls primarily have adverse health effects. The observation that higher intake of dicarbonyls via the diet is related to a lower risk of type 2 diabetes in fact supports the idea that dietary MGO intake has beneficial effects, although more data from different populations is needed and the exact underlying mechanisms need further investigation. This might also be the case for dietary 3-DG, as we also observed an association between 3-DG intake and lower incidence of type 2 diabetes, which did not relate to MGO intake.

### Strengths and limitations

To the best of our knowledge, this is the first study to examine health effects of dietary dicarbonyl intake in a prospective setting. This study has several strengths. First, the large study population, the large number of type 2 diabetes cases, and the prospective design of the cohort, which lends more support to potential causality of dietary dicarbonyl intake. Second, our results were robust over a wide range of sensitivity analyses. Third, the EPIC cohort includes eight European countries, elaborating on the previous studies on dietary dicarbonyl intake which only included individuals from the Netherlands [4, 5]. The multi-centric design also gives insight into the associations between dietary dicarbonyl intake and type 2 diabetes in different settings and in countries with different dietary patterns, providing various sources of dicarbonyl intakes. Fourth, the food composition database used in this study is the most extensive database to date [2]. Moreover, we extended this database by analysing dicarbonyls concentrations in 59 additional foods consumed in EPIC centres, to tailor this database to EPIC dietary data. Reassuringly, median intakes of MGO, GO, and 3-DG in this population were similar to intakes reported in a Dutch cohort (EPIC versus The Maastricht Study: MGO: 3.1 versus 3.6 mg/day. GO: 2.8 versus 3.5 mg/day. 3-DG: 12 versus 17 mg/day), and similar food groups contributed to dicarbonyl intakes in both populations [4].

Notwithstanding these strengths, it is important to consider potential sources of measurement error when interpreting the estimates of dietary dicarbonyl intake in this population. Potential measurement errors in dietary dicarbonyls intake may have occurred due to self- or interviewer-administered dietary questionnaires or errors in the dicarbonyl food composition database. Dicarbonyls in foods are mainly formed during heat-treatment, and although we took preparation methods into account in this database, certain variations in food processing and preparation remain. Since these errors are likely to be random, it will results in attenuation bias meaning that any true associations would be stronger than observed in our study. Potential measurement error in our outcome, i.e. incident type 2 diabetes, would have resulted in misclassification bias, which may bias estimates in either direction. To limit this, cases were vigorously ascertained and verified with multiple sources of evidence. Another limitation is potential reverse causation, although excluding cases diagnosed within the first years of follow-up or individuals with prevalent metabolic health issues did not change our results. Moreover, potential unmeasured and residual confounding can never be fully excluded. To limit this, we adjusted our analyses for a wide range of confounders and performed sensitivity analyses with adjustment for other dietary components such as the Mediterranean diet, healthy food groups that were also main sources of dietary dicarbonyls, and soft drinks consumption. However, in observational nutrition studies it is virtually impossible to fully disentangle the effects of a specific component from the effects of other dietary components in the food matrix [31]. In our study, coffee was the main contributor to MGO intake, and it has been previously shown that coffee consumption is associated with a lower incidence of type 2 diabetes [32]. This may suggest that the beneficial effects of coffee are, at least in part, attributable to MGO. Nevertheless, coffee also contains other bio-active compounds with known positive health effects such as polyphenols [33], and we cannot exclude the possibility that our associations between MGO intake and a lower risk of type 2 diabetes might be attributable to other compounds in coffee. However, 3-DG was also associated with lower incidence of type 2 diabetes, despite the small contribution of coffee to 3-DG intake, and this association was not attenuated by adjustment for coffee consumption.

## Conclusion

In conclusion, higher dietary intakes of the dicarbonyls MGO and 3-DG were associated with a lower risk of type 2 diabetes. Future research is needed to confirm these findings, understand the underlying biological mechanisms of these associations, and explore the presence of a hormetic effect for MGO.

## Supplementary Information

Below is the link to the electronic supplementary material.

Supplementary file1 (DOCX 292 kb)

## Data Availability

EPIC-InterAct Study data cannot be deposited publicly as these collaborative data originate from multiple research institutions across eight European countries with different legal frameworks. The authors confirm that researchers seeking the analysis dataset for this work can submit a data request to the EPIC-InterAct study central contact point by emailing interact@mrc-epid.cam.ac.uk.
