# Supplementary material for: Higher intake of dietary dicarbonyl compounds is associated with lower incidence of type 2 diabetes: European Prospective Investigation into Cancer and Nutrition (EPIC)-InterAct case-cohort study
Source: Eur J Nutr. 2026 Mar 17;65(3):98. doi: 10.1007/s00394-026-03904-0 (PMC12996025; doi:10.1007/s00394-026-03904-0)
Supplement: Supplementary file 1 — Supplementary file1 (DOCX 292 kb) [file 394_2026_3904_MOESM1_ESM.docx]

**Online supplementary material**

**1. Supplementary results**

Country-specific results

Country-specific random effects meta-analyses showed overall a significant between-country heterogeneity for associations of MGO, GO, and 3-DG intakes (MGO: I² = 71.7 %, p=0.001. GO: I² = 73.0 %, p=0.001, 3-DG: I² = 58.7 %, p=0.018, supplementary figure 2). The associations of MGO were generally inverse, except for Spain and Italy, in which MGO intake was the lowest (supplementary table 1) and non-significant associations were observed. The inverse association between MGO intake and incident type 2 diabetes was strongest in France (HR 0.73 [0.62-0.87], supplementary figure 2), potentially because France only included women and the association in the overall cohort seemed more pronounced for women (men: HR 0.93 [0.89-0.98] ; women: 0.91 [0.88-0.96], Supplementary tables 3 and 4). The associations of GO, the majority of the associations were inverse, except for Spain. For 3-DG, the association was stronger in France (HR 0.77 [0.63-0.94]) compared to other countries.

**Supplementary Tables**

**Supplementary table 1. Intakes of dicarbonyls and food groups in overall EPIC-InterAct subcohort (n=15,797) and stratified per country**

|  | **Overall (n=15,797)** | **France (n=580)** | **Italy (n=1,952)** | **Spain (n=3,513)** | **United Kingdom (n=1,298)** | **The Netherlands (n=1,462)** | **Germany (n=2,024)** | **Sweden (n=2,887)** | **Denmark (n=2,081)** |
| --- | --- | --- | --- | --- | --- | --- | --- | --- | --- |
| **Dicarbonyls** |  |  |  |  |  |  |  |  |  |
| MGO intake ^1^ | 3.4 ± 1.3 | 3.07 ± 0.91 | 2.53 ± 0.72 | 2.54 ± 0.82 | 3.64 ± 1.15 | 3.61 ± 1.03 | 3.56 ± 1.25 | 3.47 ± 1.18 | 4.64 ± 1.44 |
| GO intake | 2.9 ± 1.0 | 3.09 ± 0.92 | 2.61 ± 0.87 | 2.59 ± 0.85 | 3.49 ± 1.07 | 2.92 ± 0.82 | 3.25 ± 1.07 | 2.64 ± 0.83 | 3.33 ± 0.94 |
| 3-DG intake | 13.8 ± 10.5 | 11.42 ± 4.52 | 9.55 ± 3.93 | 8.53 ± 7.31 | 23.30 ± 20.19 | 13.08 ± 6.27 | 17.19 ± 8.96 | 14.37 ± 11.6 | 18.07 ± 7.87 |
|  |  |  |  |  |  |  |  |  |  |
| **Food groups/items** ^2^ |  |  |  |  |  |  |  |  |  |
| Coffee | 384 ± 384 | 275 ± 248 | 95 ± 66 | 123 ± 130 | 403 ± 354 | 547 ± 308 | 426 ± 344 | 493 ± 355 | 807 ± 474 |
| Cereal | 219 ± 116 | 212 ± 106 | 336 ± 161 | 208 ± 98 | 194 ± 111 | 184 ± 83 | 195 ± 81 | 206 ± 111 | 215 ± 82 |
| Fruits, nuts and seeds | 242 ± 190 | 266 ± 172 | 249 ± 208 | 327 ± 233 | 246 ± 186 | 219 ± 147 | 144 ± 95 | 181 ± 132 | 182 ± 153 |
| Vegetables | 184 ± 120 | 287 ± 135 | 183 ± 92 | 249 ± 145 | 257 ± 127 | 133 ± 52 | 126 ± 59 | 131 ± 95 | 177 ± 99 |
| Cakes and biscuits | 43 ± 46 | 36 ± 31 | 46 ± 45 | 37 ± 48 | 65 ± 60 | 32 ± 25 | 61 ± 58 | 48 ± 38 | 20 ± 21 |
| Beer | 104 ± 249 | 26 ± 76 | 24 ± 66 | 51 ± 149 | 63 ± 183 | 53 ± 175 | 182 ± 330 | 120 ± 193 | 254 ± 429 |
| Sugar and confectionary | 38 ± 32 | 40 ± 32 | 40 ± 30 | 23 ± 22 | 51 ± 43 | 42 ± 29 | 36 ± 32 | 45 ± 35 | 43 ± 32 |
| Fruit and vegetable juices | 16 ± 1 | 61 ± 92 | 34 ± 61 | 24 ± 63 | 50 ± 65 | 83 ± 97 | 176 ± 210 | 61 ± 106 | 36 ± 62 |

^1^ Mean ± standard deviation intake (mg/day), crude intakes not adjusted for energy-intake

^2^ Main food groups contributing to dicarbonyl intake

MGO: methylglyoxal, GO: glyoxal, 3-DG: 3-Deoxyglucosone

**Supplementary Table 2. HRs (95% CIs) for incident type 2 diabetes according to dicarbonyl intake, sensitivity analyses, EPIC InterAct (n=27,043)^1^**

|  | | | | **Dicarbonyl intake** | | |  | |  |  | | |  |
| --- | --- | --- | --- | --- | --- | --- | --- | --- | --- | --- | --- | --- | --- |
|  | N | Continuous (per 1 SD increase) | Q1 | | Q2 | Q3 | | Q4 | | | Q5 | | |
| **MGO intake in mg/day (median [IQR])** |  | 3.12 [2.42-4.05] | 2.16 [1.74-2.68] | | 2.84 [2.38-3.43] | 3.20 [2.70-3.94] | | 3.57 [2.92-4.34] | | | 4.33 [3.41-5.38] | | |
| **n cases/N** | 27,043 | 11,995 / 27,043 | 2,628 / 5,641 | | 2,435 / 5,444 | 2,383 / 5,394 | | 2,236 / 5,245 | | | 2,313 / 5,319 | | |
| **Model 2** | **27,043** | **0.92 (0.90, 0.95)** | **1.00** | | **0.91 (0.83, 1.00)** | **0.87 (0.79, 0.95)** | | **0.82 (0.74, 0.90)** | | | | **0.79 (0.72, 0.87)** | |
| Model 2 + other dicarbonyls | 27,043 | 0.91 (0.88, 0.94) | **1.00** | | 0.91 (0.83, 1.00) | 0.87 (0.79, 0.96) | | 0.82 (0.74, 0.91) | | | 0.80 (0.72, 0.89) | | |
| Model 2 + Mediterranean diet | 27,043 | 0.93 (0.90, 0.96) | **1.00** | | 0.92 (0.84, 1.01) | 0.87 (0.80, 0.96) | | 0.82 (0.75, 0.90) | | | 0.80 (0.73, 0.88) | | |
| Model 2 + soft drinks intake | 27,043 | 0.93 (0.9, 0.96) | **1.00** | | 0.92 (0.84, 1.00) | 0.87 (0.80, 0.96) | | 0.82 (0.75, 0.90) | | | 0.8 (0.73, 0.88) | | |
| Model 2 + hs-CRP | 25,623 | 0.93 (0.90, 0.96) | **1.00** | | 0.91 (0.82, 1.00) | 0.87 (0.79, 0.95) | | 0.83 (0.76, 0.92) | | | 0.8 (0.72, 0.88) | | |
| Model 2 + waist circumference ^2^ | 25,246 | 0.92 (0.89, 0.95) | **1.00** | | 0.89 (0.81, 0.98) | 0.85 (0.78, 0.94) | | 0.81 (0.73, 0.89) | | | 0.78 (0.71, 0.86) | | |
| Exclusion incident T2D during first 2 years of follow-up (n cases/N = 10,970/27,043)^3^ | 27,043 | 0.93 (0.90, 0.96) | **1.00** | | 0.91 (0.82, 1.00) | 0.86 (0.78, 0.95) | | 0.82 (0.75, 0.91) | | | 0.81 (0.73, 0.89) | | |
| Exclusion incident T2D during first 4 years of follow-up (n cases/N = 9,408/27,043) ^3^ | 27,043 | 0.94 (0.91, 0.97) | **1.00** | | 0.89 (0.81, 0.98) | 0.87 (0.79, 0.96) | | 0.85 (0.77, 0.93) | | | 0.84 (0.76, 0.92) | | |
| Exclusion prevalent CVD (n cases/N=7,301/19,543) | 19,543 | 0.93 (0.90, 0.97) | **1.00** | | 0.91 (0.82, 1.02) | 0.84 (0.75, 0.93) | | 0.80 (0.72, 0.89) | | | 0.85 (0.76, 0.94) | | |
| Exclusion HbA1c ≥ 6.5% (n cases/N=9,428/24,114) | 24,114 | 0.92 (0.89, 0.95) | **1.00** | | 0.91 (0.83, 1.00) | 0.85 (0.77, 0.94) | | 0.80 (0.73, 0.88) | | | 0.80 (0.72, 0.88) | | |
| **GO intake in mg/day (median [IQR])** |  | 2.79 [2.22-3.45] | 1.97 [1.61-2.44] | | 2.48 [2.11-2.97] | 2.79 [2.37-3.29] | | 3.10 [2.64-3.63] | | | 3.67 [3.15-4.35] | | |
| **n cases/N** | 27,043 | 11,995 / 27,043 | 2,757 / 5,770 | | 2,392 / 5,401 | 2,361 / 5,372 | | 2,161 / 5,170 | | | 2,324 / 5,330 | | |
| **Model 2** | **27,043** | **0.97 (0.94, 1.00)** | **1.00** | | **0.93 (0.85, 1.02)** | **0.92 (0.84, 1.01)** | | **0.83 (0.75, 0.92)** | | | **0.94 (0.86, 1.04)** | | |
| Model 2 + other dicarbonyls | 27,043 | 1.04 (1.00, 1.08) | **1.00** | | 1 (0.91, 1.09) | 1.01 (0.92, 1.12) | | 0.95 (0.85, 1.05) | | | 1.13 (1.01, 1.26) | | |
| Model 2 + Mediterranean diet | 27,043 | 0.99 (0.96, 1.02) | **1.00** | | 0.95 (0.87, 1.04) | 0.95 (0.86, 1.04) | | 0.86 (0.78, 0.96) | | | 0.99 (0.90, 1.10) | | |
| Model 2 + soft drinks intake | 27,043 | 0.97 (0.94, 1.00) | **1.00** | | 0.93 (0.85, 1.02) | 0.92 (0.84, 1.01) | | 0.83 (0.75, 0.91) | | | 0.94 (0.85, 1.03) | | |
| Model 2 + hs-CRP | 25,623 | 0.97 (0.94, 1.00) | **1.00** | | 0.95 (0.87, 1.04) | 0.93 (0.84, 1.02) | | 0.83 (0.75, 0.92) | | | 0.95 (0.85, 1.05) | | |
| Model 2 + waist circumference^2^ | 25,246 | 0.99 (0.95, 1.02) | **1.00** | | 0.94 (0.86, 1.04) | 0.93 (0.84, 1.03) | | 0.86 (0.77, 0.95) | | | 0.98 (0.89, 1.09) | | |
| Exclusion incident T2D during first 2 years of follow-up (n cases/N = 10,970/27,043) ^3^ | 27,043 | 0.97 (0.94, 1.00) | **1.00** | | 0.93 (0.85, 1.02) | 0.92 (0.84, 1.02) | | 0.83 (0.75, 0.92) | | | 0.94 (0.85, 1.04) | | |
| Exclusion incident T2D during first 4 years of follow-up (n cases/N = 9,408/27,043) ^3^ | 27,043 | 0.98 (0.95, 1.01) | **1.00** | | 0.96 (0.87, 1.06) | 0.94 (0.85, 1.03) | | 0.85 (0.76, 0.94) | | | 0.98 (0.88, 1.08) | | |
| Exclusion prevalent CVD (n cases/N=7,301/19,543) | 19,543 | 0.96 (0.93, 1.00) | **1.00** | | 0.92 (0.83, 1.02) | 0.88 (0.79, 0.98) | | 0.85 (0.76, 0.95) | | | 0.9 (0.80, 1.01) | | |
| Exclusion HbA1c ≥ 6.5% (n cases/N=9,428/24,114) | 24,114 | 0.98 (0.95, 1.02) | **1.00** | | 0.92 (0.84, 1.01) | 0.92 (0.83, 1.01) | | 0.82 (0.74, 0.91) | | | 0.97 (0.88, 1.07) | | |
| **3-DG intake in mg/day (median [IQR])** |  | 11.5 [7.80-16.8] | 7.27 [5.10-10.2] | | 9.61 [7.00-12.8] | 11.7 [8.53-15.5] | | 14.0 [10.0-18.5] | | | 19.8 [14.0-28.1] | | |
| **n cases/N** | 27,043 | 11,995 / 27,043 | 2,831 / 5,844 | | 2,431 / 5,440 | 2,432 / 5,443 | | 2,227 / 5,236 | | | 2,074 / 5,080 | | |
| **Model 2** | **27,043** | **0.93 (0.90, 0.95)** | **1.00** | | **0.94 (0.86, 1.03)** | **0.99 (0.90, 1.08)** | | **0.85 (0.77, 0.93)** | | | **0.80 (0.73, 0.89)** | | |
| Model 2 + other dicarbonyls | 27,043 | 0.93 (0.90, 0.97) | 1.00 | | 0.95 (0.86, 1.04) | 1.00 (0.91, 1.10) | | 0.87 (0.78, 0.96) | | | 0.83 (0.74, 0.92) | | |
| Model 2 + Mediterranean diet | 27,043 | 0.93 (0.90, 0.96) | 1.00 | | 0.95 (0.86, 1.04) | 1.00 (0.91, 1.09) | | 0.86 (0.78, 0.94) | | | 0.81 (0.74, 0.90) | | |
| Model 2 + soft drinks intake | 27,043 | 0.92 (0.90, 0.95) | 1.00 | | 0.94 (0.85, 1.03) | 0.98 (0.9, 1.08) | | 0.84 (0.77, 0.93) | | | 0.8 (0.73, 0.89) | | |
| Model 2 + hs-CRP | 25,623 | 0.93 (0.90, 0.96) | 1.00 | | 0.95 (0.86, 1.04) | 1.02 (0.93, 1.12) | | 0.86 (0.78, 0.94) | | | 0.81 (0.73, 0.90) | | |
| Model 2 + waist circumference ^2^ | 25,246 | 0.93 (0.90, 0.97) | 1.00 | | 0.98 (0.89, 1.07) | 1.02 (0.93, 1.12) | | 0.85 (0.77, 0.94) | | | 0.84 (0.76, 0.93) | | |
| Exclusion incident T2D during first 2 years of follow-up (n cases/N = 10,970/27,043) ^3^ | 27,043 | 0.93 (0.90, 0.96) | 1.00 | | 0.94 (0.86, 1.04) | 0.99 (0.90, 1.08) | | 0.86 (0.79, 0.95) | | | 0.81 (0.73, 0.89) | | |
| Exclusion incident T2D during first 4 years of follow-up (n cases/N = 9,408/27,043) ^3^ | 27,043 | 0.93 (0.90, 0.96) | 1.00 | | 0.96 (0.87, 1.05) | 1.00 (0.91, 1.09) | | 0.88 (0.79, 0.97) | | | 0.82 (0.74, 0.91) | | |
| Exclusion prevalent CVD (n cases/N=7,301/19,543) | 19,543 | 0.92 (0.89, 0.96) | 1.00 | | 0.96 (0.87, 1.07) | 0.97 (0.87, 1.07) | | 0.83 (0.74, 0.93) | | | 0.8 (0.72, 0.90) | | |
| Exclusion HbA1c ≥ 6.5% (n cases/N=9,428/24,114) | 24,114 | 0.93 (0.90, 0.96) | 1.00 | | 0.95 (0.86, 1.04) | 1.01 (0.92, 1.11) | | 0.84 (0.77, 0.93) | | | 0.82 (0.74, 0.91) | | |

^1^ Model 2: Adjusted for energy intake, educational level, BMI, height, physical activity, smoking status, alcohol, menopause, hormone use

Dicarbonyl intakes are standardized energy adjusted residuals. Quintiles are country-specific and based on non-cases. Analyses after additional adjustment for risk factors of T2D, or after exclusion of individuals.

^2^ residuals of waist, regressed for BMI to prevent multicollinearity

^3^ cases in first 2 or 4 years of follow-up recoded to non-cases

MGO: methylglyoxal, GO: glyoxal, 3-DG: 3-Deoxyglucosone

**Supplementary Table 3. P-values for interaction ^1^**

|  | MGO | GO | 3-DG |
| --- | --- | --- | --- |
| Sex | 0.36 | 0.01 | 0.36 |
| BMI | 0.26 | 0.04 | 0.08 |
| Waist | 0.81 | 0.27 | 0.36 |
| Smoking | 0.15 | 0.67 | 0.55 |
| Inflammation (hs-CRP) | 0.10 | 0.88 | 0.38 |
| Gamma-glutamyltransferase (categorical) ^2^ | 0.16 | 0.89 | 0.73 |
| Age (continuous) ^3^ | 0.25 | 0.82 | 0.24 |
| Kidney function (eGFR - continuous) | 0.33 | 0.07 | 0.05 |

^1^ Results are for the fully adjusted Model (Model 2), adjusted for energy intake, educational level, BMI, height, physical activity, smoking status, alcohol, menopause, hormone use

² Gamma-glutamyltransferase (GGT) score used, high or normal levels.

^3^ Age at recruitment as continuous variable. Age strata of 5 year as strata. Similar results if age is not in strata.

**Supplementary Table 4. HRs (95% CIs) for incident type 2 diabetes according to dicarbonyl intake, stratified by sex ^1^**

|  | Dicarbonyl intake |  |  |  |  |  |
| --- | --- | --- | --- | --- | --- | --- |
|  | Continuous  (per 1 SD increase) | Q1 | Q2 | Q3 | Q4 | Q5 |
| **Men (n=11,544)** |  |  |  |  |  |  |
| MGO intake in mg/day (median [IQR]) | 3.47 [2.71-4.49] | 2.51 [2.03-3.10] | 3.12 [2.61-3.76] | 3.52 [2.87-4.37] | 3.91 [3.27-4.84] | 4.95 [3.89-6.03] |
| n cases | 5947 | 1193 | 1287 | 1317 | 1130 | 1020 |
| Model 1 | 0.97 (0.93, 1.01) | 1 (reference) | 0.96 (0.86, 1.08) | 0.97 (0.87, 1.09) | 0.88 (0.79, 1.00) | 0.92 (0.82, 1.05) |
| Model 2 | 0.93 (0.89, 0.98) | 1 (reference) | 0.95 (0.83, 1.09) | 0.98 (0.86, 1.13) | 0.88 (0.77, 1.01) | 0.80 (0.69, 0.92) |
|  |  |  |  |  |  |  |
| GO intake in mg/day (median [IQR]) | 2.95 [2.36 – 3.62] | 2.19 [1.8 – 2.68] | 2.74 [2.33 – 3.25] | 3.08 [2.63 – 3.6] | 3.39 [2.93 – 3.97] | 3.99 [3.4 – 4.68] |
| n cases | 5,947 | 1,694 | 1,316 | 1,168 | 951 | 818 |
| Model 1 | 0.89 (0.86, 0.93) | 1 (reference) | 0.80 (0.72, 0.89) | 0.82 (0.73, 0.91) | 0.73 (0.65, 0.82) | 0.75 (0.66, 0.85) |
| Model 2 | 0.94 (0.89, 0.98) | 1 (reference) | 0.89 (0.78, 1.00) | 0.89 (0.78, 1.02) | 0.78 (0.68, 0.9) | 0.88 (0.77, 1.02) |
|  |  |  |  |  |  |  |
| 3-DG intake in mg/day (median [IQR]) | 14.0 [9.48-20.2] | 9.04 [6.49-12.6] | 11.4 [8.14-15.4] | 13.6 [9.70-17.8] | 16.1 [11.6-21.2] | 23.2 [17.1-31.4] |
| n cases | 8880 | 1260 | 1116 | 1225 | 1190 | 1156 |
| Model 1 | 0.88 (0.85, 0.92) | 1 (reference) | 0.84 (0.75, 0.95) | 0.87 (0.77, 0.98) | 0.75 (0.70, 0.85) | 0.68 (0.61, 0.77) |
| Model 2 | 0.91 (0.86, 0.95) | 1 (reference) | 0.93 (0.80, 1.07) | 1.05 (0.91, 1.21) | 0.82 (0.72, 0.95) | 0.76 (0.65, 0.88) |
| **Women (n=14,648)** |  |  |  |  |  |  |
| MGO intake in mg/day (median [IQR]) | 2.87 [2.22-3.72] | 1.96 [1.59-2.40] | 2.66 [2.22-3.13] | 2.97 [2.43-3.60] | 3.30 [2.65-4.00] | 4.03 [3.18-4.95] |
| n cases | 5768 | 1367 | 1073 | 1023 | 1061 | 1244 |
| Model 1 | 0.96 (0.93, 0.99) | 1 (reference) | 0.84 (0.76, 0.94) | 0.81 (0.73, 0.90) | 0.80 (0.72, 0.89) | 0.89 (0.80, 0.99) |
| Model 2 | 0.91 (0.88, 0.96) | 1 (reference) | 0.88 (0.77, 1.00) | 0.78 (0.68, 0.89) | 0.76 (0.67, 0.87) | 0.80 (0.70, 0.91) |
|  |  |  |  |  |  |  |
| GO intake in mg/day (median [IQR]) | 2.65 [2.11 – 3.29] | 1.74 [1.46 – 2.09] | 2.25 [1.93 – 2.62] | 2.58 [2.21 – 3.01] | 2.91 [2.49 – 3.36] | 3.53 [3.02 – 4.15] |
| n cases | 5768 | 988 | 1,016 | 1,140 | 1,159 | 1,465 |
| Model 1 | 0.97 (0.94, 1.01) | 1 (reference) | 0.90 (0.8, 1.01) | 0.87 (0.78, 0.97) | 0.82 (0.74, 0.92) | 0.93 (0.84, 1.04) |
| Model 2 | 1.00 (0.96, 1.05) | 1 (reference) | 1.02 (0.88, 1.17) | 0.97 (0.84, 1.12) | 0.91 (0.78, 1.05) | 1.03 (0.89, 1.19) |
|  |  |  |  |  |  |  |
| 3-DG intake in mg/day (median [IQR]) | 10.1 [6.85-14.4] | 6.44 [4.43-8.78] | 8.82 [6.31-11.4] | 10.8 [7.60-13.6] | 12.5 [8.99-16.5] | 17.3 [12.4-24.3] |
| n cases | 5768 | 1488 | 1252 | 1144 | 997 | 887 |
| Model 1 | 0.86 (0.83, 0.89) | 1 (reference) | 0.88 (0.79, 0.97) | 0.82 (0.74, 0.90) | 0.75 (0.68, 0.84) | 0.62 (0.63, 0.77) |
| Model 2 | 0.95 (0.91, 0.99) | 1 (reference) | 0.95 (0.84, 1.08) | 0.94 (0.83, 1.07) | 0.89 (0.78, 1.01) | 0.88 (0.77, 1.01) |

^1^ MGO: methylglyoxal, GO: glyoxal, 3-DG: 3-Deoxyglucosone

Model 1: crude Model, stratified by age, sex and center

Model 2: Adjusted for energy intake, educational level, BMI, height, physical activity, smoking status, alcohol, menopause, hormone use

Dicarbonyl intakes (when used as main independent variables) are standardized energy adjusted residuals

Quintiles are country-specific and based on non-cases

France was excluded because France contained only women

**Supplementary Table 5. HRs (95% CIs) for incident type 2 diabetes according to dicarbonyl intake, BMI stratified, EPIC InterAct (n=27,043) ^1^**

|  | | **Dicarbonyl intake** | | |  |  |  |
| --- | --- | --- | --- | --- | --- | --- | --- |
|  | | Continuous (per 1 SD increase) | Q1 | Q2 | Q3 | Q4 | Q5 |
| **GO intake** | |  |  |  |  |  |  |
| Underweight + normal weight combined ² (1,667/8,601) | | 0.93 (0.87, 0.99) | 1 (reference) | 0.82 (0.69, 0.98) | 0.86 (0.72, 1.03) | 0.75 (0.63, 0.91) | 0.79 (0.65, 0.95) |
| Pre-obesity (n cases/N= 5,264/11,250) | | 0.93 (0.89, 0.97) | 1 (reference) | 0.86 (0.75, 0.97) | 0.81 (0.72, 0.92) | 0.76 (0.67, 0.87) | 0.85 (0.75, 0.98) |
| Obesity Class I (n cases/N= 3,593/5,331) | | 1.01 (0.95, 1.07) | 1 (reference) | 0.92 (0.77, 1.11) | 0.96 (0.80, 1.16) | 0.89 (0.73, 1.07) | 1.04 (0.86, 1.25) |
| Obesity Class II (n cases/N= 1,111/1,432) | | 1.02 (0.91, 1.14) | 1 (reference) | 0.80 (0.57, 1.12) | 1.10 (0.77, 1.58) | 0.74 (0.51, 1.07) | 1.07 (0.75, 1.52) |
| Obesity Class III (n cases/N= 360/429) | | 1.13 (0.87, 1.46) | 1 (reference) | 3.62 (1.28, 10.26) | 1.72 (0.69, 4.30) | 4.24 (1.43, 12.52) | 1.32 (0.57, 3.05) |
|  | | | | | | | |

^1^ Results are for the fully adjusted Model (Model 2), adjusted for energy intake, educational level, BMI, height, physical activity, smoking status, alcohol, menopause, hormone use

GO intake (when used as main independent variables) is standardized energy adjusted residuals.

Quintiles are country-specific and based on non-cases

² BMI WHO categories: Underweight=13.6-18.5; Normal weight=18.5-24.9; Pre-obesity=25.0-29.9; Obesity Class I= 30.0-34.9; Obesity Class II= 35.0-39.9; Obesity Class III = 40.0-67.9

GO: glyoxal

**Supplementary Table 6. HRs (95% CIs) for incident type 2 diabetes according to dicarbonyl intake, additional adjustment for food groups, EPIC InterAct (n=27,043) ^1^**

|  | **Dicarbonyl intake** | | |  |  |  |
| --- | --- | --- | --- | --- | --- | --- |
|  | Continuous (per 1 SD increase) | Q1 | Q2 | Q3 | Q4 | Q5 |
| MGO intake in mg/day (median [IQR]) | 3.12 [2.42-4.05] | 2.16 [1.74-2.68] | 2.84 [2.38-3.43] | 3.20 [2.70-3.94] | 3.57 [2.92-4.34] | 4.33 [3.41-5.38] |
| **n cases/N** | 11,995 / 27,043 | 2,628 / 5,641 | 2,435 / 5,444 | 2,383 / 5,394 | 2,236 / 5,245 | 2,313 / 5,319 |
| **Model 2** | 0.92 (0.90, 0.95) | 1 (reference) | 0.91 (0.83, 1.00) | 0.87 (0.79, 0.95) | 0.82 (0.74, 0.90) | 0.79 (0.72, 0.87) |
| Model 2 + coffee intake | 0.98 (0.93, 1.03) | 1 (reference) | 0.94 (0.86, 1.04) | 0.92 (0.84, 1.02) | 0.89 (0.80, 0.99) | 0.91 (0.80, 1.03) |
| Model 2 + meat intake | 0.92 (0.89, 0.95) | 1 (reference) | 0.91 (0.83, 1.00) | 0.86 (0.79, 0.95) | 0.81 (0.74, 0.89) | 0.79 (0.71, 0.86) |
| Model 2 + vegetables intake | 0.92 (0.89, 0.95) | 1 (reference) | 0.91 (0.83, 1.00) | 0.87 (0.79, 1.00) | 0.81 (0.74, 0.89) | 0.79 (0.72, 0.87) |
| Model 2 + cereals intake | 0.92 (0.90, 0.95) | 1 (reference) | 0.91 (0.83, 1.00) | 0.87 (0.79, 0.95) | 0.82 (0.74, 0.90) | 0.79 (0.72, 0.87) |
| Model 2 + cakes and biscuits intake | 0.92 (0.89, 0.95) | 1 (reference) | 0.91 (0.83, 1.00) | 0.87 (0.79, 0.95) | 0.81 (0.74, 0.89) | 0.79 (0.72, 0.87) |
|  |  |  |  |  |  |  |
| GO intake in mg/day (median [IQR]) | 2.79 [2.22-3.45] | 1.97 [1.61-2.44] | 2.48 [2.11-2.97] | 2.79 [2.37-3.29] | 3.10 [2.64-3.63] | 3.67 [3.15-4.35] |
| **n cases/N** | 11,995 / 27,043 | 2,757 / 5,770 | 2,392 / 5,401 | 2,361 / 5,372 | 2,161 / 5,170 | 2,324 / 5,330 |
| **Model 2** | 0.97 (0.94, 1.00) | 1 (reference) | 0.93 (0.85, 1.02) | 0.92 (0.84, 1.01) | 0.83 (0.75, 0.92) | 0.94 (0.86, 1.04) |
| Model 2 + coffee intake | 0.98 (0.95, 1.02) | 1 (reference) | 0.95 (0.87, 1.04) | 0.94 (0.86, 1.04) | 0.86 (0.78, 0.95) | 0.98 (0.88, 1.08) |
| Model 2 + cereal intake | 0.97 (0.94, 1) | 1 (reference) | 0.93 (0.85, 1.02) | 0.92 (0.84, 1.01) | 0.83 (0.75, 0.92) | 0.94 (0.86, 1.04) |
| Model 2 + fruits, nuts, and seeds intake | 0.95 (0.91, 0.99) | 1 (reference) | 0.93 (0.84, 1.02) | 0.91 (0.82, 1) | 0.82 (0.73, 0.91) | 0.91 (0.81, 1.02) |
| Model 2 + vegetables intake | 0.97 (0.93, 1) | 1 (reference) | 0.93 (0.85, 1.02) | 0.92 (0.83, 1.01) | 0.83 (0.75, 0.92) | 0.94 (0.85, 1.04) |
| Model 2 + fruit and vegetable juices | 0.96 (0.93, 0.99) | 1 (reference) | 0.93 (0.85, 1.02) | 0.91 (0.83, 1) | 0.82 (0.74, 0.91) | 0.92 (0.83, 1.02) |
|  |  |  |  |  |  |  |
| 3-DG intake in mg/day (median [IQR]) | 11.5 [7.80-16.8] | 7.27 [5.10-10.2] | 9.61 [7.00-12.8] | 11.7 [8.53-15.5] | 14.0 [10.0-18.5] | 19.8 [14.0-28.1] |
| **n cases/N** | 11,995 / 27,043 | 2,831 / 5,844 | 2,431 / 5,440 | 2,432 / 5,443 | 2,227 / 5,236 | 2,074 / 5,080 |
| **Model 2** | 0.93 (0.90, 0.95) | 1 (reference) | 0.94 (0.86, 1.03) | 0.99 (0.90, 1.08) | 0.85 (0.77, 0.93) | 0.80 (0.73, 0.89) |
| Model 2 + cereal intake | 0.92 (0.90, 0.95) | 1 (reference) | 0.94 (0.86, 1.03) | 0.99 (0.90, 1.08) | 0.85 (0.77, 0.93) | 0.80 (0.73, 0.86) |
| Model 2 + fruits, nuts, and seeds intake | 0.92 (0.90, 0.95) | 1 (reference) | 0.94 (0.86, 1.03) | 0.99 (0.90, 1.08) | 0.85 (0.77, 0.93) | 0.80 (0.73, 0.89) |
| Model 2 + cakes and biscuits intake | 0.93 (0.90, 0.96) | 1 (reference) | 0.95 (0.87, 1.04) | 1.00 (0.92, 1.10) | 0.86 (0.79, 0.95) | 0.82 (0.74, 0.90) |
| Model 2 + beer | 0.92 (0.89, 0.95) | 1 (reference) | 0.94 (0.86, 1.03) | 0.99 (0.90, 1.08) | 0.84 (0.77, 0.93) | 0.78 (0.71, 0.87) |
| Model 2 + sugar and confectionary | 0.93 (0.91, 0.96) | 1 (reference) | 0.95 (0.87, 1.04) | 1.00 (0.92, 1.10) | 0.87 (0.79, 0.96) | 0.83 (0.75, 0.91) |

^1^ Model 2: Adjusted for energy intake, educational level, BMI, height, physical activity, smoking status, alcohol, menopause, hormone use

Dicarbonyl intakes are standardized energy adjusted residuals. Quintiles are country-specific and based on non-cases. Analyses after additional adjustment for either of the food groups.

MGO: methylglyoxal, GO: glyoxal, 3-DG: 3-Deoxyglucosone

**Supplementary Table 7. Concentrations additional food products quantified for matching the EPIC cohort FFQs ^1^**

|  | **MGO** | **GO** | **3-DG** |
| --- | --- | --- | --- |
|  | **mg/L or mg/kg** | **mg/L or mg/kg** | **mg/L or mg/kg** |
| Condensed milk | 0.35 | 0.34 | 22.53 |
| Evaporated milk | 0.70 | 0.25 | 1.99 |
| Porridge, rice, with whole milk, pasteurized | 0.02 | 0.00 | 0.59 |
| Yogurt, whole | 0.46 | 0.02 | 0.31 |
| Herring, pickled​ | 1.50 | 2.47 | 0.21 |
| Strawberries | 0.55 | 7.09 | 3.65 |
| Blueberries | 1.12 | 5.76 | 5.40 |
| Galia melon | 0.99 | 0.34 | 3.06 |
| Polenta, boiled with water | 0.05 | 0.00 | 2.95 |
| Breakfast cereals, puffed wheat | 5.08 | 7.41 | 32.28 |
| Breakfast cereals, puffed rice (Rice Crispies) | 9.77 | 9.11 | 46.49 |
| Liver spread/pâté​ | 2.28 | 0.55 | 2.20 |
| Liverwurst​ | 3.97 | 0.85 | 1.65 |
| Wiener sausage/frankfurter, boiled | 2.25 | 0.49 | 1.74 |
| Chicken, leg muscle, boiled | 3.01 | 0.13 | 0.09 |
| Chicken, leg muscle, roasted | 3.23 | 0.23 | 0.09 |
| Corn crisps | 3.78 | 2.95 | 12.92 |
| Chocolate, white | 0.58 | 0.46 | 20.86 |
| Gherkins | 0.42 | 0.48 | 6.77 |
| Maize, canned | 1.26 | 1.02 | 5.92 |
| Spinach, raw | 0.98 | 1.70 | 0.13 |
| Spinach, boiled | 0.49 | 0.38 | 0.17 |
| Tofu, boiled | 0.37 | 0.31 | 0.31 |
| Tofu, pan-fried | 0.54 | 0.59 | 0.75 |
| Soy sauce, unsweetened | 2.22 | 3.05 | 26.33 |
| Cheese, feta | 0.35 | 0.00 | 0.12 |
| Cheese, Camembert | 0.79 | 0.44 | 0.10 |
| Cheese, Emmentaler | 0.05 | 0.00 | 0.01 |
| Yogurt, semi-skimmed, vanilla | 0.49 | 0.05 | 4.77 |
| Instant coffee powder​, prepared | 0.79 | 0.57 | 0.86 |
| Rum, dark | 0.14 | 0.09 | 1.20 |
| Herring, raw | 1.22 | 1.51 | 0.32 |
| Nectarine | 0.56 | 2.04 | 4.59 |
| Coconut, dried | 0.57 | 0.93 | 6.91 |
| Rhubarb | 0.50 | 0.87 | 21.39 |
| Watermelon | 0.37 | 0.38 | 2.65 |
| Pita bread, plain, white | 1.28 | 0.69 | 4.97 |
| Beans, red kidney, canned | 0.89 | 1.77 | 1.26 |
| Bacon rashers (pan-fried)​ | 4.70 | 1.62 | 2.91 |
| Roast beef, cold cut​ | 1.90 | 0.57 | 0.73 |
| Lamb, pan-fried | 4.37 | 0.63 | 1.45 |
| Lamb, roasted | 3.69 | 0.28 | 1.74 |
| Rice cake/cracker (from puffed rice), with sea salt | 3.60 | 2.25 | 12.85 |
| Chocolate praline, orange crème filling | 1.17 | 3.33 | 26.41 |
| Tomato, raw | 0.57 | 1.45 | 8.19 |
| Garlic, raw | 17.68 | 1.44 | 12.71 |
| Garlic, roasted | 15.25 | 22.06 | 32.72 |
| Red cabbage, raw | 0.61 | 1.78 | 3.47 |
| Red cabbage, boiled | 0.50 | 0.83 | 3.63 |
| Butter | 0.02 | 0.06 | 0.05 |
| Almonds, unroasted | 1.06 | 1.87 | 3.29 |
| Almonds, roasted | 1.65 | 2.83 | 6.09 |
| Tova caramel desert sauce | 3.02 | 1.85 | 90.67 |
| Espresso - from restaurant | 11.47 | 1.28 | 26.92 |
| Herring, pickled, sweet and sour, without sweetener | 0.85 | 1.43 | 2.16 |
| Kellogg's Frosties | 13.79 | 18.27 | 111.03 |
| Hazelnuts | 1.25 | 2.05 | 2.84 |
| Chestnut puree, canned | 0.63 | 1.01 | 7.32 |
| Pasta, boiled, wholegrain | 1.60 | 1.52 | 4.49 |

^1^ MGO: methylglyoxal, GO: glyoxal, 3-DG: 3-Deoxyglucosone, FFQs: Food Frequency Questionnaires


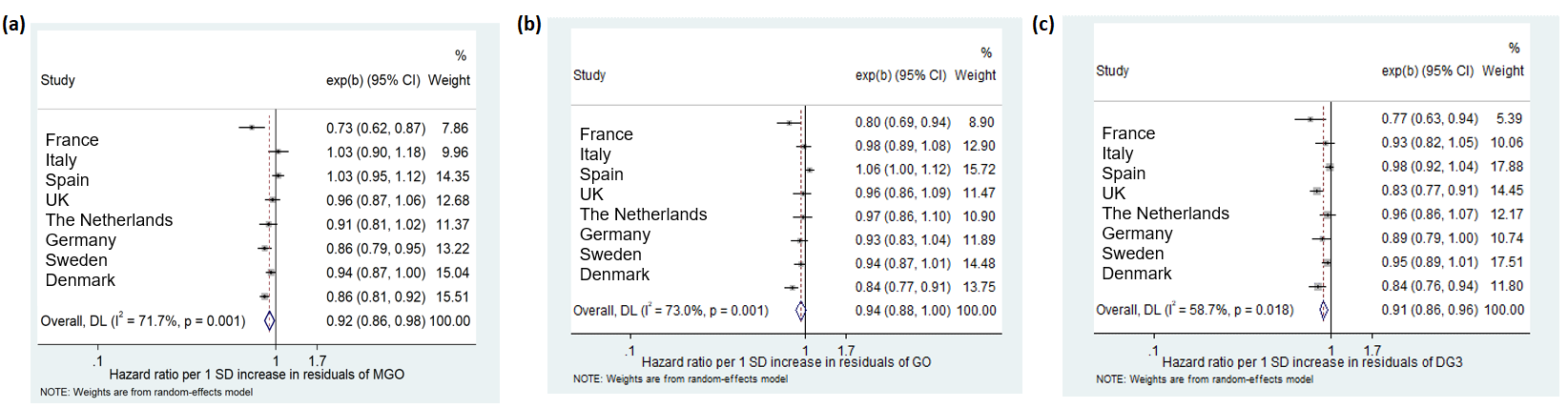


**Supplementary Figure 2. HRs (95% CIs) for incident type 2 diabetes according to dicarbonyl intake, EPIC InterAct (n=27,043), pooled estimates per country ^1^**

(a) MGO intake as main independent variable, (b) GO intake as main independent variable, (c) 3-DG intake as main independent variable

^1^ Model 2: Adjusted for energy intake, educational level, BMI, height, physical activity, smoking status, alcohol, menopause, hormone use

Dicarbonyl intakes are standardized energy adjusted residuals.

MGO: methylglyoxal, GO: glyoxal, 3-DG: 3-Deoxyglucosone
